# Iron status predicts cognitive test performance of primary school children from Kumasi, Ghana

**DOI:** 10.1371/journal.pone.0251335

**Published:** 2021-05-19

**Authors:** Afua Afreh Mantey, Reginald Adjetey Annan, Herman Erick Lutterodt, Peter Twumasi

**Affiliations:** 1 Department of Laboratory Technology, Kumasi Technical University, Kumasi, Ghana; 2 Department of Biochemistry and Biotechnology, College of Science, Kwame Nkrumah University of Science and Technology, Kumasi, Ghana; 3 Department of Food Science and Technology, College of Science, Kwame Nkrumah University of Science and Technology, Kumasi, Ghana; 4 National Sports Authority, Ghana; Addis Ababa University, ETHIOPIA

## Abstract

**Background:**

Good nutritional status of school-aged children is crucial in achieving improved cognition. The objective of this study was to assess the relationship between nutritional status and cognition of school-aged children in the Kumasi metropolis, Ghana.

**Methods:**

389 children were selected from ten government-owned schools. Socio-demographic and anthropometric data were collected. Blood samples were collected and analyzed for nutrients levels. Dietary intakes were assessed using food frequency questionnaire and previous day’s nutrients intake. Cognition test was performed using the Ravens Colored Progressive Matrix (RCPM).

**Results:**

Mean age of participants was 8.9±1.4 years, mean RCPM score was 17.9±5.4. More girls scored RCPM below the 40^th^ percentile (45.5%) than boys (33.7%), while mother’s level of education significantly associated with RCPM percentiles of the children (p = 0.037). Four dietary patterns were generated from food frequency data, and scores of the second pattern, depicting vegetables, non-fruits, bread and cereals, showed a weak negative correlation (r = -0.132, p = 0.026) with previous day’s dietary zinc intake. Cognitive status did not vary by anthropometric and dietary patterns. More anemic (54.4%) than non-anemic (33.3%) children were below the 40^th^ RCPM percentile. Mean previous day’s intake for folate (p<0.001), vitamin B_6_ (p = 0.018), iron (p<0.001), and zinc (p = 0.001) differed significantly between the cognitive test score percentiles of the children. Spearman rank correlation showed weak positive associations between RCPM score and hemoglobin (r = 0.246, p = 0.003) and serum ferritin (r = 0.176, p = 0.036). Binary regression analysis showed anemic children (aOR = 0.4; 95%CI = 0.2–0.8, p = 0.014), compared with non-anemic had decreased odds, while boys, compared with girls had increased odds (aOR = 2.0 95%CI = 1.0–4.0, p = 0.035) for scoring above the 50^th^ RCPM percentile.

**Conclusions:**

Iron status, especially hemoglobin levels, correlated with the cognitive performance of school-aged children in the metropolis. Thus nutritional strategies aimed at reducing iron deficiency anemia are needed.

## Introduction

Cognitive performance in school-aged children is influenced by several factors, including nutrition [1–3]. Cognition represents a complex set of higher mental functions served by the brain and includes attention, memory, thinking, learning, and perception [4,5]. There is much development of higher executive cognitive functions during the school-age period, such as reasoning and abstract thinking. Cognitive development is of particular importance to school-aged children because it is crucial for acquiring factual knowledge and behavioral and social skills. It is also relevant for learning and good academic performance [6,7].

Though the relationship between nutrition and cognition is complex, nutrient composition and meal patterns can have immediate or long-term beneficial or adverse effects on cognitive abilities in children [8,9]. Poor nutrition status negatively affects cognitive development and performance among school children. Thus, nutritionally deficient children are more likely not to perform well in school and score poorly in cognitive tests due to slow memory recall and attention problems than well-nourished children [10–12]. Nevertheless, improving nutritional status subsequently improves cognition and academic performance among school children [13,14]. Iron, iodine, zinc, B-vitamins, flavonoids, and long-chain fatty acids, such as docosahexaenoic acid (DHA), and polyunsaturated fatty acids are nutrients that are essential for brain or cognitive development and function, especially in children [1,3]. Iron plays a vital role in brain function and is involved in brain energy production, neurotransmitter synthesis, and myelination. Thus, brain areas responsible for cognitive outcomes are also sensitive to iron deficiencies [8,9,15,16]. Cross-sectional, longitudinal, and intervention studies show that iron deficiency with or without anemia has adverse effects on cognitive development and performance in children. There is a preponderance of evidence demonstrating that anemic children have poorer cognition and school achievement than non-anemic children [17–19]. Zinc deficiency may also impair cognition development by altering attention, activity, and motor development [20]. Iodine is essential for developing the central nervous system through neurogenesis, axon and dendrite growth, and myelination. Evidence suggests that iodine deficiency negatively affects cognitive performance and intelligence in children [21]. Apart from the effect of single nutrients, multiple micronutrients interact synergistically to maintain or improve cognitive function and performance. However, in most developing countries, school-aged children are deficient in one or more of these nutrients mainly due to low dietary intakes of nutrients, low bioavailability of nutrients due to parasitic infections, and anti-nutritional inhibitors such as phytates [13,22].

Malnutrition results in stunting, wasting, and underweight among children. Conversely, in recent times overweight/obesity is also becoming a concern among school-aged children. Cross-sectional studies indicate associations between delayed growth or stunting, intelligence quotient (IQ), and school performance among school children. For example, a study among Southeast Asian children indicated that undernourishment and non-verbal IQ were significantly associated in 6–12-year-old children [23]. Another study in Ethiopia among school children suggested that weight-for-age and cognition influenced their academic performance [24]. Meanwhile, significant impairment in cognitive functions such as attention, retention, and intelligence was observed among school adolescents with high body mass index (BMI) [25].

There is a paucity of information on school-aged children’s nutritional status in Ghana, particularly in urban areas. Few studies in rural areas suggest that stunting, anemia, and nutrient deficiencies are prevalent among school-aged children [26–28]. Implicit in this observation is the likelihood of decreased cognitive performance among school-aged children in Ghana, which can affect their academic performance and economic potential in the future. A study among Ghanaian school-aged children reported that dietary micronutrient intakes were inadequate in most of these children. However, there was no significant association with their cognitive test performance [27,28]. More studies are needed to investigate the relationship between cognitive test performance and nutrition status among school-aged children in Ghana. The current study investigates the association between nutritional status and cognitive performance among school-aged children in an urban setting in Ghana.

## Materials and methods

### Study area and population

The cross-sectional study was conducted within the Kumasi metropolis of the Ashanti Region of Ghana, with a population density of 9822 people/km^2^. The Kumasi metropolis is the district capital of the Ashanti region, and it is one of the 30 administrative districts. The metropolis has a high school rating, and it is an important education center. There are about 203 government-owned basic or primary schools as well as privately owned ones.

### Sample size and sampling procedure

The children chosen for the study attended government-owned primary schools in the metropolis. The sample size (n) based on the population of children in government-owned primary schools in the metropolis was calculated using the Yamane formula (1967).

Yamane’s formula for sample size determination is mathematically given as

$$n_{2}=\frac{N_{2}}{1+N_{2}e^{2}}=\frac{67475}{1+67475{\left(0.05\right)}^{2}}=397.64\approx 398$$

Where *n* is the sample size, *N* = population, and *e* is the sampling error usually given as 0.05 (5%). The total population of primary school-aged children in government schools within the Kumasi metropolis was obtained from the Kumasi Metropolitan Education Directorate. The total population obtained was 67475. Using the formula, a sample size of 398 children was to be recruited for the study. The education directorate has grouped Government-owned schools within the metropolis under 30 educational circuits. The schools were listed, and systematic random sampling was used to select every 5^th^ circuit. Seven (7) circuits were thus selected. Ten schools were selected conveniently from the 7circuits. The children were selected by cluster sampling from the ten schools. The sample of children selected from each school was dependent on the population of children within the age of inclusion (6-11years). School children were randomly selected from their classrooms to be part of the study. Parents and guardians of 389 pupils consented to participate in the study. All children selected were seemingly healthy with no physical handicap or apparent mental disorders. Children who were physically or mentally unhealthy or challenged were excluded.

### Ethical considerations

Ethical approval was obtained from the Committee on Human Research Publication and Ethics (CHRPE) of the Kwame Nkrumah University of Science and Technology, KNUST, (Kumasi, Ghana) (Reference: CHPRE/AP/080/17). Parents and guardians of the pupils provided written informed consent before data collection. Pupils whose parents or guardians did not consent were excluded from participating in the study. The Kumasi Metropolitan Education Directorate of the Ghana Education Service gave approval, and the heads of the selected basic schools also permitted for their schools to be included in the study.

### Socio-demographic data collection

Information on their socio-demographic profile and maternal education was collected using a pre-tested structured questionnaire.

### Dietary assessments

A food frequency questionnaire (FFQ) was used to assess the dietary intakes of the children. The food frequency questionnaire consisted of forty-one food items, grouped into eight food groups. The frequency of intake was categorized into ’never’, ’1–2 times a month’, ’1–2 times a week’, ’3–4 times a week’, and ’5 times or more a week or daily’. The previous day’s dietary nutrient intake was also assessed using the 24- hour dietary recall method. The children were asked to describe the type and amount of food and all beverages consumed during the day preceding the interview. Handy measures, food models, and photographic food atlas were used to identify and determine quantities and weights of portions of foods consumed by the participants. The portions in household measures were converted into their grams equivalent, and the composition of nutrients was analyzed with the nutrient analysis template designed by the University of Ghana, Food Science, and Nutrition Department, Accra, which contains macronutrient and micronutrient levels in Ghanaian foods [29].

### Anthropometric measurements

Anthropometric measurements were all carried out in duplicates using standard methods by a trained research assistant. Height was measured barefooted using a Seca 214 stadiometer, and results recorded to the nearest 0.1cm. Weight was measured using a digital weighing scale to the nearest 0.1kg with the children in standard school uniform without shoes, socks, or any heavy clothing. Height-for-age z-score (HAZ), weight-for-age z-score (WAZ), and BMI-for-age z-score (BAZ) were calculate using World Health Organization (WHO) AnthroPlus software and compared with reference data according to the WHO 2006 population [30]. Children below negative two standard deviations (−2SD) according to the WHO median for WAZ, HAZ, and BAZ were considered wasted, stunted, or thin, respectively. Normal WAZ, HAZ, and BAZ, were defined as z-scores greater than or equal to -2SD

### Collection of blood sample

Five (5) ml non fasting venous blood was drawn from each child by trained phlebotomists. Two (2) ml of the blood sample was immediately transferred into ethylenediaminetetraacetic acid (EDTA) tube to be used for hematological analysis, and 3ml was placed in a serum separator tube for biochemical analysis. The samples were stored immediately in an icebox with ice packs and then transported to the clinical laboratory within 3 hours of collection. Blood in the serum separator tube was centrifuged for 15 minutes at 4000 rpm. Aliquots of the serum were transferred into microcentrifuge tubes/Eppendorf tubes and stored at -80°C at the Komfo Anokye Teaching Hospital, Kumasi. Hemoglobin concentration was measured the same day on arrival at the laboratory on the whole blood using an automated hematological analyzer. Anemia was defined as Hemoglobin level <11. 5 g/dl. Infection was defined as C-reactive protein (CRP) > 5mg/L. Serum ferritin was analyzed by a sandwich enzyme-linked immunosorbent assay (ELISA) technique. Iron stores were considered depleted when ferritin was <15 mg/L. Serum zinc using non fasting blood was analyzed by a colorimetric method using an automated chemistry analyzer. Zinc deficiency was defined by plasma zinc concentration< 65 μg/dl in children <10 years; < 70 μg/dl in girls ≥ 10 years, < 74 μg/dl in boys ≥ 10 years. Results of children with CRP levels > 5mg/L indicating infection or inflammation were excluded.

### Cognitive test assessment

The Raven’s Colored Progressive Matrices (RCPM) was used for the assessment of cognitive performance among school children. It is designed for young children ages 5–11 years [31]. The Raven’s Colored Progressive Matrices (RCPM) comprises three sets of twelve problems that measure the ability to solve problems and reasoning by analogy. It is a non-verbal test that has been used extensively as a culturally fair test of intelligence. Non—verbal intelligence and fluid cognitive abilities are responsible for memory performance, processing, problem-solving, and reasoning, essential for school-age children in learning and academic achievement [32].

The test was administered in all the schools between 8 am to 11 am in a near distraction-free environment by well-trained research assistants. The test was administered individually to the participants following the Ravens Colored Progressive Matrix (RCPM) administrative procedures by using the RCPM printed booklet and scored using the score sheet. The examinee identifies the missing component from six multiple-choice options in a series of figural patterns starting from Set A to Set AB to Set B while the examiner ticks the options chosen by the examinee on the score sheet. The score sheets are marked using the scoring scheme. The total score for the test is 36. However, the score for each examinee is simply the correct responses made.

### Data analysis

The Ravens test scores obtained by the participants were converted into percentiles. The pupils were grouped into those who scored below the 40^th^ percentile, those from the 40^th^– 65^th^ percentile, and those above the 65^th^ percentile. Analysis of variance (ANOVA) was used to determine the difference between the different cognitive score groups. Descriptive analysis was conducted to obtain frequencies and percentages. Associations between cognitive test score percentiles, socio-demographics, and the other nutritional variables (anthropometry, blood parameters) were examined using a chi-square test and regression analysis. Binary logistic regression analysis was further performed to determine predictors of cognitive performance. Principal Component Analysis (PCA) was used to simplify the FFQ data. PCA aims to simplify the correlations between numerous interrelated variables. Using the PCA method, we identified patterns from the FFQ to represent usual food intake patterns. Four dietary patterns were determined using the PCA. Scores for each of the patterns were derived for each participant. These scores were used as continuous variables to determine the bivariate correlation coefficient between cognitive scores and dietary patterns scores. We also converted the scores into two categories: lower and upper halves, and compared them with percentiles of the cognitive test scores for chi-square analysis. The data were analyzed using Statistical Package for Social Sciences version 25 (SPSS IBM Inc).

## Results

### Cognitive test performance by socio-demographic status

Table 1 presents the socio-demographic profile of the pupils by their cognition test performance. The mean age of the children was 8.9±1.4 years. Significant associations were observed between maternal literacy (p = 0.037) and the cognition performance percentile. In terms of gender, more females (45.5%) than males (33.7%) were below the 40^th^ percentile for cognitive score, while more males (47.9%) than females (29.1%) had cognitive test scores above the 65^th^ percentile. No significant association was observed between cognition test scores of children living with both parents or not.

**Table 1:** Socio-demographic characteristics of study participants and cognition test performance.

SD variable	Total	Cognition performance percentile N(%)			P-value
		Below 40^th^	40-65th	above 65th	
**Mean Age**	**8.9±1.4**	**8.5±1.4**	**9.0±1.3**	**9.1±1.3**	**<0.001**
**Mean RCPM score**	**17.9±5.4**	**12.8±2.8**	**18.0±0.8**	**23.4±3.3**	**<0.001**
**Gender**					
Girls	220(56.6)	100(45.5)	56(25.5)	64(29.1)	**0.001**^¥^
Boys	169(43.4)	57(33.7)	31(18.3)	81(47.9)	
**Living with**					
Both parents	216(55.5)	84(38.9)	50(23.1)	82(38.0)	0.796^¥^
Other^1^	173(45.5)	73(42.2)	37(21.4)	63(36.4)	
**Mother’s education**					
Basic/Primary	89(27.4)	37(41.6)	17(19.1)	35(39.3)	**0.037**^†^
Secondary	51(15.7)	15(29.4)	8(15.7)	28(54.9)	
Tertiary	7(2.2)	4(57.1)	3(42.9)	0(0.0)	
Illiterate	178(54.8)	73(41.0)	46(25.8)	59(33.1)	

Other^1^- mother or father only, other relatives or non-relatives. Data are presented as frequency (percentage), SD- Sociodemographic,

^†^- Fisher’s exact P value,

^¥^- Chi-square P-value. Bold values are significant at p<0.05.

### Patterns of dietary intake

The summarized Table 2 indicates dietary patterns of food groups consumed by participants generated by Principal Component Analysis. The patterns were grouped according to correlation coefficient factor ≥ 0.3 for positive and negative values. Before performing the principal component analysis, the suitability of the data for factor analysis was assessed. Four patterns were shown from the scree plot to reflect the main dietary intake patterns from the FFQ data. The principal component analysis showed four components with eigen values exceeding 1, explained as a percentage of variances: 18.3%, 5.7%, 4.6%, and 4.2%. Pattern 1 was the most consumed pattern, followed by pattern 2, and pattern 4 had the least consumed foods. Pattern 1 reflected varied foods consumption and showed significant correlation coefficients for grains and cereals, dairy products, legumes, fruits, vegetables and egg, fish and meat-based foods, and fat and oil. In contrast, pattern 2 recorded significant correlations for vegetables, some cereal bread-based, non-processed foods, and non-fruits foods. Pattern 3 recorded some grain rice, egg-based, some oil, non-vegetables, and non-fruit foods. Pattern 4 shows vegetables, some grain cassava, non-processed foods, and non-fruits foods.

**Table 2:** Dietary pattern of food groups consumed by participants.

Dietary pattern	Pattern 1	Pattern 2	Pattern 3	Pattern 4
% Variances	18.3	5.7	4.6	4.2
Food sources	All Varied food groups	Vegetables, some cereals bread-based non-processed foods and non-fruits	some grain rice, egg-based, some oils, non-vegetable and non-fruits	Vegetables, some grain, non-processed foods, and non- fruits
**Correlation coefficient**				
**Grain and cereals**				
Millet				
Maize	0.397			
Bread		0.311		
Rice			0.386	
Instant noodles/pasta				
Yam	0.373			
Plantain	0.448			
Cocoyam	0.503			
Gari	0.395			
Cassava	0.342			0.34
Sweet potato				
**Dairy foods**				
Milk	0.347			
Yogurt	0.558			
**Vegetables**				
Cocoyam leaves (*Kontomire*)	0.499			0.489
*“Ayoyo”*	0.405	0.314	-0.363	0.501
Carrots	0.484	0.675		
Tomatoes	0.472	0.421		
Cabbage	0.408	0.641		
**Legumes**				
Beans	0.39			
Groundnuts	0.542			
**Animal protein**				
Fish	0.354			
Chicken	0.401			
Beef	0.417			
Egg	0.417		0.363	
Sausage	0.323			
**Fruits**				
Orange	0.426	-0.298		
Banana	0.618			
Watermelon	0.506		-0.300	
Apple	0.579		-0.300	
Mango	0.309			
Pawpaw	0.549	-0.302	-0.359	
Pineapple	0.516			
Pear	0.525			
**Processed/Snack foods/drinks**				
Popcorn	0.473			-0.353
Plantain chips	0.565			
Fruit juice	0.525	-0.311		
Hibiscus drink	0.494			
Pastry	0.351			
**Fat and oil**				
Margarine	0.397			
Palm oil	0.392			
Vegetable oil			0.569	

Table 3 presents the Spearman correlations and p-values between dietary intake patterns generated by PCA and the previous day’s intake of selected nutrients from the 24-hour dietary recalls. The correlation analysis between scores of the patterns of dietary intake and means of the previous day’s nutrient intake showed no significant correlations for patterns 1, 3, and 4. On the other hand, the second pattern showed a weak negative but statistically significant associations with dietary zinc (r = -0.12, p = 0.026).

**Table 3:** Spearman rank correlations and statistical significance of dietary patterns and the previous day’s nutrient intake.

		Iron	Zinc	Vitamin B_12_	Vitamin B_6_	Folate
**Pattern 1 N = 285**	Correlation Coefficient	-0.008	-0.033	-0.028	-0.015	0.017
	**P-value**	0.895	0.583	0.662	0.798	0.775
**Pattern 2 N = 285**	Correlation Coefficient	-0.101	-0.132*	0.055	-0.074	-0.028
	**P-value**	0.089	**0.026**	0.386	0.216	0.642
**Pattern 3 N = 285**	Correlation Coefficient	0.017	0.047	0.000	0.056	0.042
	**P-value**	0.777	0.425	0.995	0.347	0.486
**Pattern 4 N = 285**	Correlation Coefficient	-0.022	-0.010	-0.026	-0.040	-0.013
	**P-value**	0.707	0.871	0.681	0.505	0.832

p-values are significant at p<0.05.

### Cognitive test performance by dietary patterns, anthropometrics, iron status, and serum nutrients

Table 4 shows the comparisons of proportions and means of children who obtained cognition test scores below the 40^th^, within 40-65^th^ and above the 65^th^ percentiles by anthropometric, hematological, serum iron and zinc status and by previous day’s nutrients intake. For anthropometrics, cognition status did not significantly vary by weight-for-age, height-for-age, or BMI-for-age categories. The association between the cognition test score and hemoglobin status was significant (p = 0.004). 54.4% of anemic children were below the 40^th^ percentile for cognitive test scores compared to 33.3% for non-anemic children. The majority of the non-anemic children (42.5%) had relatively higher cognition test scores (> 65^th^ percentile) for cognition, whilst for those with anemia, 27.8% had relatively higher cognition scores (> 65^th^ percentile). Of the serum nutrients assessed, ferritin was not significantly associated with cognitive test performance (p = 0.375). In contrast, serum zinc showed a significant association (p<0.001), with the majority of the children with low serum zinc (45.5%) scoring above the 65^th^ percentile for the cognitive test. For the four dietary patterns, the proportions of participants within the three cognitive performance score categories did not vary compared to lower and upper halves of scores for the four dietary patterns. There were significant differences between the mean previous day’s dietary intake of folate (p = <0.001), vitamin B_6_ (p = 0.018), iron (p = <0.001), and zinc (p = 0.001) between the pupils who scored below the 40^th^ percentile, between 40-65^th^ percentile and those who scored above the 65^th^ percentile.

**Table 4:** Relationship between cognition status and nutrition status.

			Cognition Test Scores		
Variable	Total	Below 40^th^	40-65th	above 65th	P-value
**Anthropometrics**					
**Weight-for-age**					
Underweight	11(3.3)	4(36.4)	4(36.4)	3(27.3)	0.143^†^
Normal	293(86.7)	135(42.7)	72(22.8)	109(34.5)	
overweight/obese	34(10.1)	1(12.5)	1(12.5)	6(75.0)	
**Height-for-age**					
Stunted	11(2.8)	5(45.5)	0(0.0)	6(54.5)	0.173^†^
Normal	378(97.2)	152(40.2)	87(23.0)	139(36.8)	
**BMI-for-age**					
Underweight	11(2.8)	4(36.4)	3(27.3)	4(36.4)	0.859^†^
Normal	350(90.0)	141(40.3)	80(22.9)	129(36.9)	
overweight/obese	28(7.2)				
**Biochemistry and hematology**					
**Hemoglobin (g/dl)**					
Anemic	79(25.7)	43(54.4)	14(17.7)	22(27.8)	**0.004**^¥^
Not anemic	228(74.3)	76(33.3)	55(24.1)	97(42.5)	
**Serum zinc (μg/L)**					
Low	22(7.3)	76(30.9)	58(23.6)	112(45.5)	**<0.001**^†^
Normal	280(92.7)	19(73.1)	6(23.1)	1(3.8)	
**Serum Ferritin(μg/L)**					
Low	19(7.3)	9(47.4)	5(26.3)	5(26.3)	0.375^†^
Normal	243(92.7)	85(35.0)	55(22.6)	103(42.4)	
**Patterns of dietary intake**					
**Pattern 1 score**					
Lower half	142	66(46.5)	37(26.1)	39(27.5)	0.348
Upper half	143	65(45.5)	29(20.3)	49(34.3)	
**Pattern 2 score**					
Lower half	142	62(43.7)	37(26.1)	43(30.3)	0.500
Upper half	143	69(48.3)	29(20.3)	45(31.5)	
**Pattern 3 score**					
Lower half	142	66(46.5)	29(20.4)	47(33.1)	0.501
Upper half	143	65(45.5)	37(25.9)	41(28.7)	
**Pattern 4 score**					
Lower half	142	63(44.4)	35(24.6)	44(31.0)	0.807
Upper half	143	68(47.6)	31(21.7)	44(30.8)	
**Mean previous day’s dietary micronutrients intake**					
Folate, μg	315 ± 280	238.4 ±161.7	354.8 ±309.8	375 ± 338	<0.001*
Vitamin B_6_, mg	1.38 ± 0.7	1.27 ± 0.54	1.5 ± 0.76	1.4 ± 0.7	0.018*
Vitamin B_12_, μg	1.67±1.7	1.56 ± 1.5	1.76 ± 1.8	1.72 ± 1.9	0.605*
Iron, mg	10.3± 5.8	8.73 ± 4.3	11.91 ± 6.7	11.01 ± 6.0	<0.001*
Zinc, mg	6.72 ±3.4	5.93 ± 2.86	7.39 ± 3.9	7.16 ± 3.7	0.001*

Data are presented as frequencies (percentage). Anthropometrics were measured in z score,

^†^- Fisher’s exact P value,

^¥^- Chi-square P-value. Bold values are significant at p<0.05.

*p-value (ANOVA) for the difference in mean previous day’s dietary nutrient intake between cognitive score groups in percentiles.

### Partial correlations between RCPM test score and nutritional parameters

As showed in Table 5, age-adjusted partial correlations showed significant positive correlations between Raven’s cognition test scores and hemoglobin (r = 0.246; p = 0.003), serum ferritin (r = 0.176; p = 0.036), while a negative correlation was observed between cognition test score and serum zinc (r = -0.145). Dietary patterns, weight-for-age, height-for-age, and BMI-for-age z scores were not significantly associated with cognition test scores in the age-adjusted bivariate analysis.

**Table 5:** Association between anthropometric, biochemical, dietary pattern and cognition test scores.

Variable	Cognition test score	
	r	P-value
WAZ	0.097	0.247
HAZ	0.138	0.100
BAZ	0.030	0.720
Hemoglobin	0.246	**0.003**
Serum zinc	-0.145	0.084
Serum ferritin	0.176	**0.036**
Pattern 1	-0.041	0.627
Pattern 2	-0.040	0.636
Pattern 3	0.004	0.960
Pattern 4	-0.060	0.476

Controlling for age of participants, r- Correlation co-efficient, WAZ- Weight-for-age, BAZ- BMI-for-age, HAZ- Height-for-age, p-value significant at p<0.05.

### Predictors of RCPM score

Table 6 presents the odds ratio for variables by cognitive test performance above the 50^th^ percentile. The boys had significantly higher odds (aOR = 2.0; 95% CI 1.0–4.0; p = 0.035) of obtaining a cognitive test score above the 50^th^ percentile compared to the girls. Children who were anemic (aOR = 0.4, 95% CI 0.2–0.8; p = 0.014) had significantly decreased odds of having cognitive test scores above the 50^th^ percentile compared to non-anemic children. Again, those with low serum ferritin (aOR = 0.4; 95%CI 0.1–1.5; p = 0.188) also had decreased odds of having cognitive test scores above the 50^th^ percentile though not significant. However, children with low serum zinc had higher odds (aOR = 3.6; 95%CI 1.0–13.6; p = 0.056) of scoring above the 50^th^ percentile in the cognitive test compared to those with normal levels of serum zinc, though not statistically significant.

**Table 6:** Predictors of cognition test above the 50^th^ percentile.

Variable	Cognition test performance above 50th percentile		
	Adjusted OR (aOR)	95%CI (lower-Upper)	P-value
**Gender**			
Boys	2.0	1.1–4.0	**0.035**
Girls	Ref		
**Age (years)**			
6	2.292	0.3–20.7	0.460
7	1.048	0.2–4.8	0.952
8	0.809	0.2–3.0	0.754
9	0.835	0.2–3.1	0.788
10	0.367	0.1–1.3	0.115
11	Ref		
**Mother’s education**			
Basic/Primary	0.4	0.1–1.2	0.091
Tertiary	0.1	0.1–1.0	**0.052**
Illiterate	0.7	0.2–1.6	0.287
Secondary	Ref		
**Hemoglobin status**			
Anemic	0.4	0.2–0.8	**0.014**
Not anemic	Ref		
**Serum zinc**			
Low	3.6	1.0–13.6	0.056
Normal	Ref		
**Serum ferritin**			
Low	0.4	0.1–1.5	0.188
Normal	Ref		

Adjusted for the education level of participants, meals intake per day, OR- Odds ratio, CI- Confidence Interval, p-value significant at p<0.05.

## Discussion

The present study reports the relationship between nutritional status and cognitive performance assessed using RCPM among selected school-aged children in the Kumasi metropolis. RCPM is used to measure non-verbal intelligence and fluid cognitive abilities, influenced mainly by nutritional status [33]. This study found that serum ferritin and hemoglobin status significantly correlated with cognition test scores, with hemoglobin status being the main predictor of cognition. It was observed that children who were anemic and those with low serum ferritin levels had lower odds of scoring above the 50^th^ percentile in the cognition test even with adjustment for potentially confounding variables such as grade level and the number of meals per day. There is strong evidence that iron deficiency is linked to cognitive impairment and poor academic performance among school children. This may be due to its direct impact on brain structure and function or behavioral changes particularly in anemic children. Though some findings were equivocal, it is mostly observed that children with learning difficulties and those who performed poorly in cognitive tests had iron deficiency with or without anemia [15,17,34,35]. Iron deficiency anemia is capable of causing cognitive impairments related to attention, concentration, and intelligence [18]. A study among 8-year-old school children in Brazil observed that the children with learning difficulties had significant lower hemoglobin and serum ferritin levels than those with no learning difficulty [36]. An earlier study to elucidate the effects of hemoglobin and serum ferritin levels on cognition suggested that children with low ferritin levels but high hemoglobin concentrations had significantly high cognitive function [19]. In this study, serum zinc negatively correlated with cognitive performance among the school children. Nevertheless, findings from observational and interventional studies in school-aged children suggest that though zinc intake and status may benefit cognitive performance, the evidence is limited and inconsistent [37]. For instance, while available zinc intake improved cognitive function among primary school pupils in Kenya [38], another study found no association between zinc intake and cognition [39].

HAZ, BAZ, and WAZ, which are indicators of nutrition status, are associated with cognitive development and academic performance in children. Stunting is a prevalent nutritional problem that has been particularly linked to poor cognition in children in numerous studies [40,41]. A study among Southeast Asian school-aged children reported that children with low HAZ, WAZ, and BAZ were more likely to have a below-average IQ [42]. Another study in Ethiopia observed that HAZ was significantly associated with high mathematics scores though no significant association was observed between WAZ, BAZ, and academic performance of the school children [24]. In this present study, no significant association was observed between HAZ, WAZ, and BAZ and cognition status. This could be due to the relatively low prevalence of stunting (2.8%), wasting (3.3%) and thinness/underweight (2.8%) observed, as the study was conducted among children in an urban setting where malnutrition is not much of a problem compared to those in rural settings. Similar findings were observed in a study to assess cognitive function and associated factors among school-aged children in urban Ethiopia [43].

Numerous studies suggest that dietary intake of specific nutrients and ingredients impacts cognitive function. While some studies have investigated the effects of specific nutrients on cognition, other studies have investigated the effects of dietary patterns and behaviors such as consumption of breakfast or consumption of fast foods on consumption [3,44]. Principal Component Analysis has been used to describe patterns of dietary intakes and relate these to outcome variables. In this study, when the relationship between the cognition and dietary patterns obtained from the FFQ were explored, none of the dietary patterns correlated with cognition. However, it was observed that for all the nutrients considered, the mean previous day’s nutrient intake was relatively low for the school children who obtained cognitive test scores below the 40^th^ percentile compared to those who obtained scores above the 40^th^ percentile. Implicit in this observation is that the mean previous day’s intake of iron, folate, zinc, vitamins B_6_, and B_12_ was related to their cognitive test scores. Though the mean previous day’s nutrients intake was not used to estimate their usual nutrients intake, it may give an indication of their dietary intake status of the nutrients. Evidence from studies on dietary micronutrient intake and cognition remains equivocal. A study in Ghana among school children found no association between dietary micronutrient intake and cognition [28]. However, other studies in Kenya and Korea among children found that dietary micronutrient intakes were associated with cognitive performance [38,44]. Further studies are thus required to elucidate the influence of dietary micronutrient intakes on school children’s cognitive performance.

This study had some limitations. Firstly, the design effect was not considered in calculating and adjusting the sample size for the study. The school children were interviewed directly on the previous day’s dietary intakes and dietary patterns using the FFQ. Thus biases in portion size estimation and frequency of consumption of specific foods were possible. The authors also recognize that the FFQ and 24-hr dietary recall for the previous day’s dietary intake may not be the most reliable method for this age group through the use of visual portion estimates like household handy measures and photographic food atlas during the data collection helped participants recall food portions consumed and reduce bias. Again the usual nutrients intake of the school children could not be estimated by the use of a single 24-hr recall, which was used to estimate their previous day’s nutrients intake. The study did not assess the effect of inhibitors such as phytates on iron absorption. This is also a limitation to the study.

## Conclusions

Hemoglobin levels was observed as the predictor of cognition performance among the school children in this study. Children with low hemoglobin levels had reduced odds of obtaining cognitive test scores above the 50^th^ percentile. This finding suggests that much attention needs to be given to the iron status of school children in urban settings such as the Kumasi metropolis to prevent iron deficiencies with or without anemia and improve cognitive outcomes. A significant negative correlation observed between serum zinc and cognitive performance in this study coupled with inconsistent outcomes in other studies indicates that further research is needed to consider the impact of zinc on cognition among school-aged children. Again, further studies are recommended for school-aged children in rural settings where stunting and underweight with its effects are a major problem.

## Supporting information

S1 File Survey questionnaire. (PDF)
