# Supplementary material for: Iron status predicts cognitive test performance of primary school children from Kumasi, Ghana
Source: PLoS One. 2021 May 19;16(5):e0251335. doi: 10.1371/journal.pone.0251335 (PMC8133497; doi:10.1371/journal.pone.0251335)
Supplement: S1 File — (PDF) [file pone.0251335.s001.pdf]

BASELINE STUDY OF NUTRITIONAL STATUS AND COGNITIVE PERFORMANCE AMONG SCHOOL-AGED CHILDREN (6-11 years) IN KUMASI METROPOLIS.

BASELINE SURVEY QUESTIONNAIRE

DATE: / / **(dd/mm/yy)** **ID NUMBER**

1. INTERVIEWERS NAME: ………………………………………………
2. NAME OF SCHOOL: ……………………………………………………………….
3. SCHOOL CODE: ………………………………………………………………….
4. SCHOOL PART OF SCHOOL FEEDING PROGRAM.

YES = 1

NO = 2

5. DISTRICT : KUMASI METRO

CHILD'S PROFILE

6. NAME OF PUPIL: ……………………………………………………………………
7. DATE OF BIRTH: / / (dd/mm/yr) AGE: ………..…………….
8. GENDER: **FEMALE = 1** **MALE = 2**

9. CLASS/STAGE: **1** **2** **3**

10. ETHNICITY:

AKAN = 1

GA = 2

EWE = 3

HAUSA/FRAFRA/DAGOMBA (NORTHENER) = 4

FAMILY AND SOCIOECONOMIC DATA

11. WHO DO YOU LIVE WITH?

Both Parents = 1
Mother only = 2
Father only = 3
Other = 4

(***IF THE RESPONDENT DOES NOT LIVE WITH MOTHER, FATHER OR BOTH PARENTS, SKIP TO QUESTION 21***)

12. PARENTAL MARRIED STATUS:

Live together =1
Widow(er) = 2
Divorced = 3
Other = 4

13. MOTHER'S NAME: ………………………………………………

14. CAN YOUR MOTHER READ AND /OR WRITE? **Yes = 1 No = 2 Don't Know =3**

15. EDUCATIONAL STATUS: (MOTHER)

Basic/Primary = 1
Secondary = 2
Tertiary = 3
Illiterate = 4
Don't Know = 5

16. MOTHER'S OCCUPATION: …………………………………………………………..

(Please tick and specify where possible)

Employment (Government/ Private) =1
Farming = 2
Trading = 3
Artisan works = 4
Unemployed = 5
Other = 6
Don't know = 7

17. FATHER'S NAME: …………………………………………………………

18. CAN YOUR FATHER READ AND /OR WRITE? **Yes = 1 No = 2 Don't Know = 3**

19. EDUCATIONAL STATUS:

Basic = 1
Secondary = 2
Tertiary = 3
None = 4
Don't Know = 5

20. FATHER'S OCCUPATION ..

Employment (Government/ Private) =1
Farming = 2
Trading = 3
Artisan works = 4
Unemployed = 5
Other = 6
Don't know = 7

21. WHO DO YOU CURRENTLY LIVE WITH?

Relative = 1

Non- Relative = 2

22. CAN YOUR GUARDIAN READ AND /OR WRITE? **Yes = 1 No = 2 Don't Know = 3**

23. EDUCATIONAL STATUS OF GUARDIAN

Basic =1
Secondary = 2
Tertiary = 3
Illiterate = 4
Don't Know = 5

24. WHAT WORK DOES YOUR GUARDIAN DO?

(Specify the option chosen in the space(s) provided above and specify where possible)

Employment (Government/ Private) =1
Farming = 2
Trading = 3
Artisan works = 4
Unemployed = 5
Other = 6
Don't know = 7

25. HOW MANY SIBLINGS DO YOU LIVE WITH ***(Child excluded)***?

26. HOW MANY PERSONS LIVE IN THE HOUSEHOLD ***(Child included)***?

27. PLACE OF RESIDENCE: ………………………………………………………………..

28. RESIDENCE TYPE OF RESIDENCE:

Own House = 1
Family/Compound House = 2
Rented = 3
Other = 4

MORBIDITY HISTORY

29. HAVE YOU HAD DIARRHOEA IN THE LAST 2 WEEKS? *(Diarrhoea: 3 or more watery or loose/liquid stool in 24 hrs)*

Yes = 1
No = 2

30. HAVE YOU HAD HEADACHE IN THE LAST 2 WEEKS?

Yes = 1
No = 2

31. HAVE YOU BEEN ILL WITH A FEVER AT ANY TIME IN THE LAST 2 WEEKS?

Yes = 1
No = 2

32. HAVE YOU HAD A **PERSISTENT** COUGH IN THE LAST 2 WEEKS?

Yes = 1
No = 2

33. HAVE YOU BEEN ILL WITH VOMITING IN THE LAST 2 WEEKS**?**

Yes = 1
No = 2

DIETARY PATTERN

34. HOW MANY TIMES DO YOU EAT EACH DAY? **1 , 2 , 3 , 4** ***(Please circle)***

35. WHICH OF YOUR MEALS DO YOU SOMETIMES SKIP? **Breakfast = 1 Lunch = 2 Supper = 3 None = 4**

36. DO YOU HAVE BREAKFAST DAILY BEFORE SCHOOL STARTS?

Yes = 1
No = 2
Sometimes = 3

37. DO YOU EAT THE FOOD PROVIDED BY SCHOOL FEEDING PROGRAM OR CANTEEN? :

Yes = 1
No = 2
Sometimes = 3

38. WHAT TYPE OF SALT IS USED FOR COOKING IN YOUR HOME?

IODATED = 1

NON IODATED = 2

DON'T KNOW = 3

FOOD FREQUENCY

Food Source	Frequency of Consumption				
	**Almost Daily (More than 5 times a week)**	**3-4 times a week**	**1-2 times a week**	**Occasionally (Once a month)**	**Never**
**Grains and Cereals**					
Millet (Hausa)					
Corn/Maize: (Porridge, Banku, Kenkey)					
Bread					
Rice					
Wheat					
Instant Noodles / Pasta					
**Roots and Tubers**					
Yam (Fried/Boiled)					
Plantain					
Cocoyam					
Cassava					
Sweet Potato					
**Dairy Products**					

Milk					
Yogurt					
Cheese					
**Green Leafy Vegetables**					
Kontonmire					
“Ayoyo”					
Other:					
**Other Vegetables**					
Carrot					
Tomato					
Cabbage					
**Legumes and Nuts**					
Beans (Soya Beans)					
Ground nuts					
**Animal Protein**					
Fish					
Chicken					
Mutton/Beef/ Liver/Pork					
Egg					
Sausage (Processed)					
**Fruits**					
Orange					

Banana					
Watermelon					
Apple					
Mango					
Pawpaw					
Pineapple					
Pear					
Other……………………					
**Snacks**					
PopCorn					
Plantain Chips					
Juice Drinks (packed)					
Sobolo					
Pastry (Cakes, Pies, Doughnuts etc)					
Other: …………………					
**Fats & Oils**					
Margarine/Butter					
Palm Oil					
Vegetable oil					

Now I would like to ask you some questions about the food(s) you ate yesterday at home and outside home (school) . I know this is sometimes hard to remember, but please give me the best answer you can.

24 HOUR FOOD RECALL

	**TIME**	**DETAILS OF FOOD OR BEVERAGE CONSUMED**	**AMOUNT/ SERVING SIZE**	**LEFT OVER**	**COMMENTS**
**BREAKFAST**<br><br>Did you have anything to eat or drink at breakfast yesterday?<br><br>What did you have?				YES ☐<br><br>NO ☐	
**MORNING SNACK**<br>**(BREAK)**<br>Did you eat or drink anything on the way to school or during the morning break at school?				YES ☐<br><br>NO ☐	

What did you have?					
	**TIME**	**DETAILS OF FOOD OR BEVERAGE CONSUMED**	**AMOUNT/ SERVING SIZE**	**AMOUNT/ SERVING SIZE**	**COMMENTS**
**LUNCH (AFTERNOON BREAK)**<br>Did you have anything to eat or drink at lunch break yesterday?<br><br>What did you have?				YES ☐<br><br>NO ☐	

**LATE AFTERNOON LUNCH/SNACK**<br><br>Did you eat or drink anything on your way home from school?<br><br>Did you eat or drink anything before your evening meal?				YES ☐<br><br>NO ☐	
**SUPPER**<br><br>Did you have an evening meal yesterday?<br><br>What did you have?				YES ☐<br><br>NO ☐	
**EVENING SNACK**				YES ☐<br>☐	

Did you have anything else to eat or drink after your evening meal, before you went to bed?<br><br>What did you have?				NO	

ANTHROPOMETRY ASSESSMENT

WEIGHT : **W1** ______________ (Kg) **W2** _____________ (Kg)

HEIGHT : H1 ________________ (cm) H2 ______________ (cm)

WAIST CIRCUMFERENCE : ____________(cm) ______________(cm)

BIOCHEMICAL ASSESSMENTS

SAMPLE ID NO : BLOOD [ ]

URINE [ ]

DATE OF SAMPLE COLLECTION : [ ]

**Biochemical Variable**		
Haemoglobin		
Haematocrit		

Serum Ferritin		
Serum Zinc		
Serum Retinol		
Serum Folate/B12		
Urine Iodine		

RESULT CODE: [ ]

Completed=1
Incomplete=2

Reason, in case the data collection was not completed :
……………………………………………………………………………………
……………………………………………………………………………………
……………………………………………………………………………………
